# Selenium Protects Mouse Hypothalamic Cells from Glucocorticoid-Induced Endoplasmic Reticulum Stress Vulnerability and Insulin Signaling Impairment

**DOI:** 10.3390/antiox12020526

**Published:** 2023-02-20

**Authors:** Katlyn J. An, Ashley N. Hanato, Katherine W. Hui, Matthew W. Pitts, Lucia A. Seale, Jessica L. Nicholson, Pamela Toh, Jun Kyoung Kim, Marla J. Berry, Daniel J. Torres

**Affiliations:** 1 Department of Cell and Molecular Biology, John A. Burns School of Medicine, University of Hawai‘i, Honolulu, HI 96813, USA; 2 Pacific Biosciences Research Center, School of Ocean and Earth Science and Technology, University of Hawai‘i, Honolulu, HI 96822, USA

**Keywords:** corticosterone, endoplasmic reticulum stress, glucocorticoid, hypothalamus, insulin, selenium

## Abstract

The use of glucocorticoid medications is known to cause metabolic side effects such as overeating, excess weight gain, and insulin resistance. The hypothalamus, a central regulator of feeding behavior and energy expenditure, is highly responsive to glucocorticoids, and it has been proposed that it plays a role in glucocorticoid-induced metabolic defects. Glucocorticoids can alter the expression and activity of antioxidant enzymes and promote the accumulation of reactive oxygen species. Recent evidence indicates that selenium can counter the effects of glucocorticoids, and selenium is critical for proper hypothalamic function. This study sought to determine whether selenium is capable of protecting hypothalamic cells from dysfunction caused by glucocorticoid exposure. We treated mHypoE-44 mouse hypothalamic cells with corticosterone to study the effects on cellular physiology and the involvement of selenium. We found that corticosterone administration rendered cells more vulnerable to endoplasmic reticulum stress and the subsequent impairment of insulin signaling. Supplementing the cell culture media with additional selenium alleviated endoplasmic reticulum stress and promoted insulin signaling. These findings implicate a protective role of selenium against chronic glucocorticoid-induced hypothalamic dysfunction.

## 1. Introduction

Glucocorticoids (GCs) are a class of steroid hormones that are released by the adrenal gland as a result of the activation of the hypothalamic-pituitary-adrenal (HPA) axis, which comprises the endocrine component of the physiological “stress response” of an organism [1]. Due to their anti-inflammatory and immune-suppressive properties, GCs are widely prescribed in humans for the treatment of various conditions, including asthma, osteoarthritis, and auto-immune disorders, and patients must oftentimes take GCs for 6 months or longer [2]. Prolonged use of GCs can cause a variety of detrimental side effects, including metabolic impairments like overeating, excess weight gain, and hyperglycemia [3]. Accumulating evidence indicates that the brain plays a significant role in mediating the metabolic disturbances caused by long-term GC exposure [4]. Such a role is likely to involve the hypothalamus, a major regulator of feeding and energy metabolism. Hypothalamic dysfunction caused by redox imbalance, inflammation, and endoplasmic reticulum (ER) stress can lead to adverse metabolic effects, such as overeating and hyper-adiposity [5]. Excessive GC action within the brain could worsen these conditions, as GCs have been shown to impair redox-regulating enzymes, leading to the accumulation of reactive oxygen species (ROS), oxidative damage, and ER stress [6,7]. Additionally, GCs are known to have negative effects on insulin sensitivity [8].

The essential micronutrient selenium (Se) is known to affect energy homeostasis, and altered Se status has been associated with metabolic disturbances like type II diabetes mellitus (T2DM) [9,10]. The highly reactive Se atom is incorporated into redox-regulating selenoproteins in the form of the 21st amino acid, selenocysteine (Sec). The brain relies heavily on antioxidant Se to function properly, as it has a high rate of metabolism and is, therefore, particularly vulnerable to oxidative damage and dysfunction [11]. Recent studies have highlighted the importance of Se in supporting the ability of the hypothalamus to properly regulate energy homeostasis [12]. Moreover, the redox actions of selenoproteins have the potential to alter neuronal activity dynamics [13]. In addition to helping to prevent oxidative insults, Se plays a role in alleviating ER stress [14].

To investigate the interactions between Se, GCs, and ER stress, we used a mouse hypothalamic cell line. The aim of the present study was to determine if selenium is effective in preventing glucocorticoid-induced hypothalamic cell dysfunction. We hypothesized that (1) GCs would make the cells more susceptible to ER stress and the associated insulin resistance, and (2) that Se supplementation would mitigate these effects. We treated mHypoE-44 cells with corticosterone (CORT), the main active GC in mice, and observed a potentiation of ER stress that impaired insulin signaling in a manner that was reversible by Se supplementation. Our findings implicate Se as a key factor involved in GC-induced hypothalamic dysfunction.

## 2. Materials and Methods

### 2.1. Antibodies and Chemicals

The following primary antibodies were used for western blotting: anti-Protein kinase B (Akt) monoclonal antibody [D9E] (1:1000; Cell Signaling, #4060, RRID:AB_2315049; Danvers, MA, USA), anti-Phosphorylated protein kinase B (Phospho-Akt) polyclonal antibody (1:1000; Cell Signaling, #9272, RRID:AB_329827), anti-C/EBP homologous protein (CHOP) monoclonal antibody [L63F7] (1:1,000; Cell Signaling, #2895, RRID:AB_2089254), and anti-β-actin monoclonal antibody [8H10D10] (1:5000; Cell Signaling, #3700, RRID:AB_2242334). Secondary antibodies consisted of highly cross-adsorbed IRDye-conjugated secondary antibodies from LI-COR (1:10,000).

The following chemicals were used in experiments: CORT (≥ 98.5%; Millipore Sigma, #27840; Burlington, MA, USA), Humulin N isophane insulin suspension U-100 (Eli Lilly; Indianapolis, IN, USA), and tunicamycin (≥98%; Millipore Sigma, #T7765). The concentrations of CORT used are comparable to serum CORT levels induced by an acute stress event in rodents (1 µM is equal to 346.46 ng/mL) [15]. Tunicamycin induces the misfolded protein response by preventing the N-glycosylation of glycoproteins, thereby preventing their transport from the ER, and the concentration used (1 µM) is within the range of that commonly used in vitro to induce *Chop* expression [16].

### 2.2. Cell Culture

Mouse hypothalamic cells (mHypoE-44 purchased from Cellutions, #CLU136, RRID:CVCL_D457, Missoula, MT, USA)) were cultured in Dulbecco’s modified Eagle medium (Corning, NY, USA, #10-013-CV) containing 10% fetal bovine serum (HyClone, #SH30406.02HI, lot DE27192264 containing 165 nM Se, Logan, UT, USA), and 1% penicillin/streptomycin (ThermoFisher, #10378016, Waltham, MA, USA). Cell culture media contained 100 nM Se, unless otherwise stated, in the form of sodium selenite (Sigma, #214485, Burlington, MA, USA), and cells were kept in a humidified incubator with 5% CO_2_ at 37 °C. This is an immortalized neuronal cell line derived from mouse embryonic hypothalamic primary cells and expresses both the glucocorticoid receptor (GCR) and the insulin receptor (cedarlanelabs.com, accessed on 24 January 2023). Cells were plated on 6-well plates for experiments, and media were changed every 48 h. For a 7-day experiment, this amounted to CORT being administered a total of 4 times. Experimental conditions and timelines are described in more detail in the figure captions.

### 2.3. Western Blotting

Cells were lysed with CelLytic Mammalian Tissue Buffer (Millipore Sigma, #C3228) containing a protease/phosphatase inhibitor (Cell Signaling, #5872), centrifuged, and the supernatant collected. Proteins (80 µg per sample) were loaded onto 4–20% gradient polyacrylamide TGX gels (BIO-RAD, Cat# 5671094; Hercules, CA, USA), separated by size via electrophoresis, and transferred to an Immobilon-FL 0.45 µm pore polyvinylidene difluoride membrane (Millipore Sigma, # IPFL00010). Membranes were blocked using Intercept (PBS) Blocking Buffer (LI-COR, #927-70001; Lincoln, NE, USA), after which primary antibodies were added and membranes allowed to incubate at 4 °C overnight. The next day, infrared fluorescent secondary antibodies were added for 30 min, and blots were imaged using the Odyssey XF Imaging System (LI-COR) and analyzed using Image Studio software (LI-COR, RRID:SCR_015795).

### 2.4. Statistical Analysis

A two-way analysis of variance (ANOVA) was used to compare experimental groups. Each experiment consisted of four conditions, each with a sample size of four. Tukey’s multiple comparisons test was used as a *post-hoc* test in all cases. All data were analyzed and plotted using GraphPad Prism version 7. All data shown in graphs is represented as the mean ± the standard error of the mean, and sample sizes are indicated in figure captions. Significance was determined with a *p* value < 0.05.

## 3. Results

### 3.1. Corticosterone Potentiates Endoplasmic Reticulum Stress Induction

**Figure 1:**
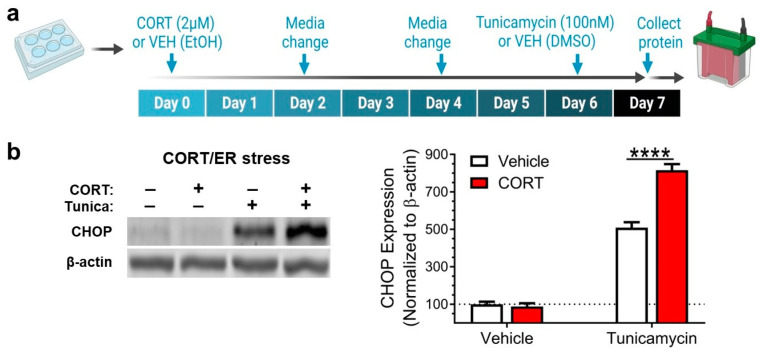
Corticosterone exposure for 7 days makes mHypoE-44 cells more vulnerable to endoplasmic reticulum stress. (**a**) Experimental design: cells were exposed to corticosterone (CORT) or vehicle (VEH; ethanol, EtOH) for 7 days, and tunicamycin was added for the last 21 h prior to protein harvest. (**b**) Pre-exposure to CORT increased the endoplasmic reticulum (ER) stress response induced by tunicamycin, measured as (C/EBP Homologous Protein) CHOP protein levels (two-way ANOVA: interaction: F(1,12) = 42.7, *p* < 0.0001; Tukey’s post-hoc: VEH/tunicamycin vs. CORT/tunicamycin, **** *p* < 0.0001; n = 4 for all groups). All values shown are mean ± standard error of the mean.

We began by exposing mHypoE-44 cells to CORT via a total of four media changes over a span of seven days (168 h) and adding the ER stress inducer tunicamycin for 21 h before cell lysis and protein collection for western blot (Figure 1a). We found that CORT-exposed cells showed a greater increase in the ER stress marker CHOP (C/EBP homologous protein) caused by tunicamycin application than vehicle-treated (VEH) control cells (Figure 1b).

### 3.2. Corticosterone Exacerbates the Insulin Signaling Impairment Caused by Endoplasmic Reticulum Stress

**Figure 2:**
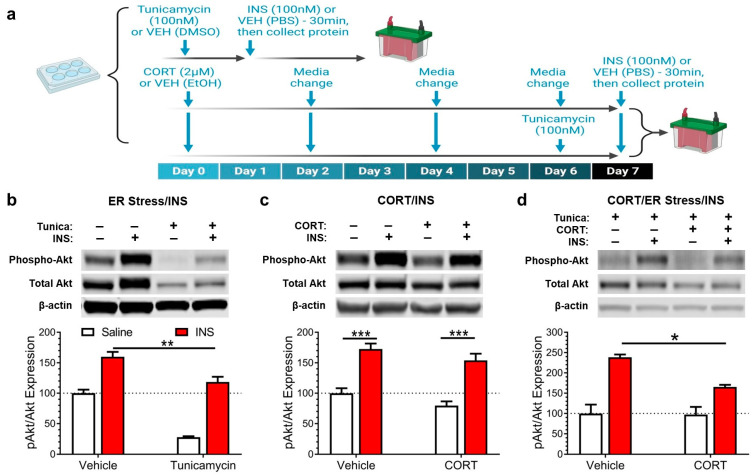
Endoplasmic reticulum stress impairs insulin signaling in mHypoE-44 cells, an effect amplified by pre-exposure to corticosterone. (**a**) Experimental design: Panel b—Endoplasmic reticulum (ER) stress was induced by applying tunicamycin for 21 h, and insulin (INS) was added for the last 30 min prior to cell lysis/protein extraction. Panel c: the INS challenge was also performed after 7 days of corticosterone (CORT) application. Panel d: 7 days of CORT application, followed by tunicamycin and the INS challenge. (**b**) ER stress induction blunted the response to INS (two-way ANOVA: interaction: F(1,12) = 5.5, *p* = 0.04, Tunicamycin: F(1,12) = 74.1, *p* < 0.0001; Tukey’s post-hoc: VEH/INS vs. Tunicamycin/INS, ** *p* = 0.004; n = 4 for all groups). (**c**) CORT exposure for 7 days slightly reduced the response to INS (two-way ANOVA: CORT: F(1,12) = 4.8, *p* = 0.049; Tukey’s: VEH/Saline vs. VEH/INS *** *p* = 0.0005, CORT/Saline vs. CORT/INS *** *p* = 0.0004; n = 4 for all groups). (**d**) Pre-exposure to CORT worsened the INS signaling impairment caused by ER stress induction (two-way ANOVA: interaction: F(1,12) = 5.4, *p* = 0.04; Tukey’s: VEH/INS vs. CORT/INS, * *p* = 0.02; n = 4 for all groups). All values shown are mean ± standard error of the mean.

In order to establish the physiological impact of CORT on mHypoE-44 cells, we investigated the effects on insulin signaling. To determine if similar effects occur as a result of CORT exposure, we treated our cells with insulin (Humulin) for 30 min, just prior to cell lysis (Figure 2a). We first confirmed that tunicamycin-induced ER stress attenuates insulin signaling (Figure 2b). Then, we determined that CORT alone does not have an effect on the response to insulin (Figure 2c). Finally, we found that the impairment of insulin signaling caused by tunicamycin was greater in cells exposed to CORT compared to VEH controls (Figure 2d).

### 3.3. Selenium Supplementation Prevents Corticosterone from Exacerbating Endoplasmic Reticulum Stress and Impairing Insulin Signaling

**Figure 3:**
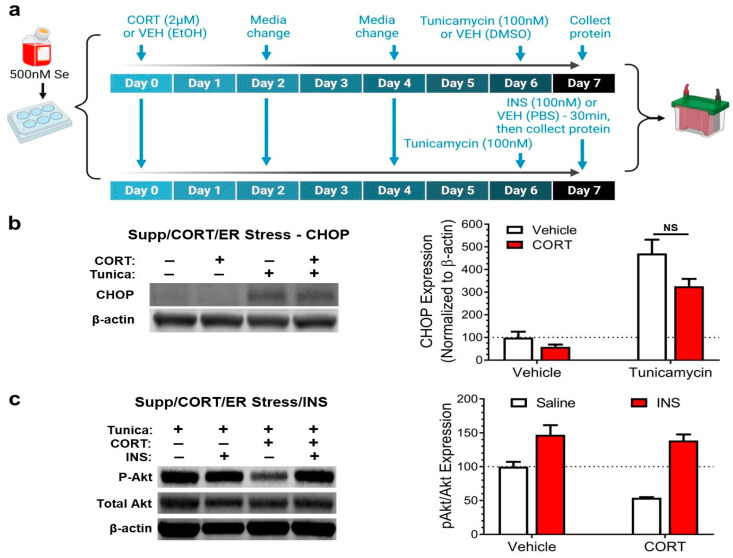
Supplementation with selenium has a restorative effect on endoplasmic reticulum stress and insulin signaling in corticosterone-exposed mHypoE-44 cells. (**a**) Experimental design: panel b: cells were treated with selenium (Se) supplementation (500 nM Se in media) in conjunction with either corticosterone (CORT) or vehicle (VEH; ethanol, EtOH) exposure, followed by endoplasmic reticulum (ER) stress induction using tunicamycin or addition of VEH (dimethyl sulfoxide, DMSO); panel c: cells were treated with Se supplementation in conjunction with either CORT or VEH, then all cells were exposed to tunicamycin, followed by either insulin (INS) or VEH (phosphate-buffered saline, PBS). (**b**) Culturing cells in media containing 500 nM Se prevented CORT from decreasing the potentiation of ER stress (two-way ANOVA: interaction: F(1,12) = 2.0, *p* = 0.2; Tukey’s: VEH/tunicamycin vs. CORT/tunicamycin: *p* = 0.07; n = 4 for all groups). (**c**) Supplementation with Se prevented CORT from worsening the impairment of INS signaling caused by ER stress (two-way ANOVA: CORT: F(1,12) = 8.9, *p* = 0.1; Tukey’s: VEH/INS vs. CORT/INS, *p* = 0.9; n = 4 for all groups). All values shown are mean ± standard error of the mean.

Thus, we next tested whether supplementing mHypoE-44 cells with extra Se in the culture media would mitigate the effects of CORT (Figure 3a). Supplementation with Se prevented CORT from exacerbating tunicamycin-induced ER stress (Figure 3b). The inhibition of insulin signaling caused by the combination of CORT and tunicamycin was also prevented by Se supplementation (Figure 3c).

## 4. Discussion

**Figure 4:**
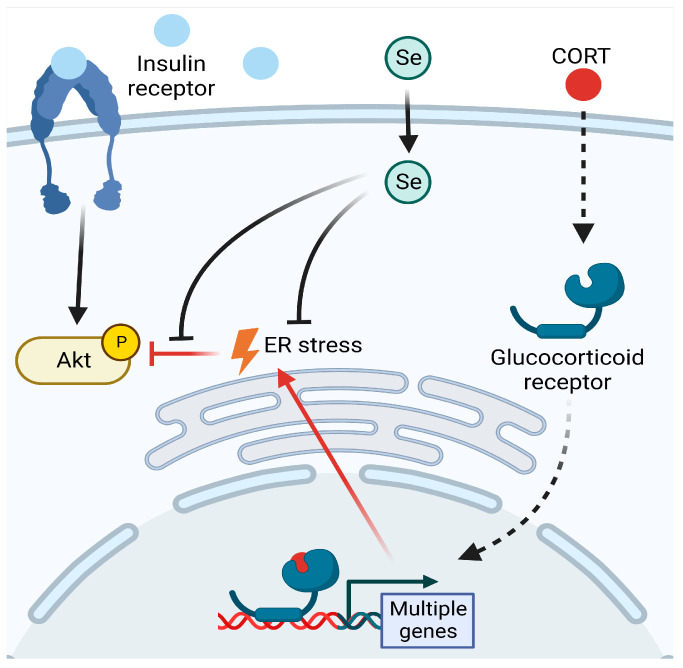
Proposed model of the ability of selenium to protect insulin signaling from being impaired by chronic corticosterone exposure in mHypoE-44 hypothalamic cells. Upon binding insulin, the insulin receptor initiates an intracellular signaling cascade that involves the activation (via phosphorylation) of protein kinase B (AKT). Endoplasmic reticulum (ER) stress limits the phosphorylation of AKT, an effect that is exacerbated by chronic exposure to corticosterone (CORT). Supplementation with additional selenium (Se) is able to prevent CORT from exacerbating ER stress and, subsequently, inhibiting insulin signaling. Red lines highlight the pathological effects of chronic CORT.

Life stress has been associated with an increased risk for diabetes and obesity in humans, and the negative impact on the ER caused by chronic stress on the cellular level has been proposed as a contributing factor. Using mouse hypothalamic mHypoE-44 cells, we have found that CORT potentiates ER stress induction. CORT exposure also worsens the inhibitory effect on insulin signaling caused by ER stress. Interestingly, these deficits are reversible with Se supplementation. The molecular interactions involved in these findings are summarized in Figure 4. These data provide new insight on the crosstalk between Se metabolism and GC action and the impact on neuronal physiology.

Previous work has established a relationship between GC action and ER stress. While some studies have suggested a protective effect against ER stress [17,18], others have indicated that GCs can worsen or even induce ER stress by themselves when delivered either (a) chronically or (b) acutely and at high concentrations [19,20]. Likewise, we found that multiple exposures to CORT over a 7-day span increased the susceptibility of mHypoE-44 cells to tunicamycin-induced ER stress. Although we found no evidence of CORT causing ER stress by itself, it did intensify the CHOP expression induced by tunicamycin. Expression of the transcription factor CHOP is induced by ER stress and subsequently promotes the expression of pro-apoptotic genes [21]. There are multiple ER stress signaling pathways, and activation of the pathways involving ATF4 and ATF6 leads to CHOP induction, which, in turn, upregulates pro-apoptotic factors [21].

Multiple studies have depicted a causal role for ER stress in the development of insulin resistance in hepatic, skeletal, and adipose cells [22,23,24]. A growing body of evidence also implicates ER stress in mediating the neurological damage and dysfunction caused by metabolic disease, including the associated weakening of hypothalamic leptin sensitivity [25]. The impact of ER stress on hypothalamic insulin signaling, however, has only more recently begun to come to light [26]. For example, ER stress-inducing palmitate application can impair insulin signaling in mHypoE-44 cells [27]. In our study, we similarly found that ER stress induced by tunicamycin inhibits insulin signaling in this hypothalamic cell line. Moreover, we have determined that pre-exposure to CORT can amplify this effect. Our work, therefore, identifies ER stress as a mediating event through which GCs can impair insulin signaling in hypothalamic cells.

Se supplementation has demonstrated restorative effects in various models with disruptions to selenoprotein expression and Se utilization [28,29,30]. Importantly, we found that providing cells with excess Se blocks the CORT-induced aggravation of both ER stress and insulin signaling. Previous literature indicates an ability of Se and selenoproteins to regulate insulin signaling [31,32,33], but the influence on hypothalamic insulin signaling remains under-investigated. Thus, the most likely explanation based on our results is that the protective effect of Se on insulin sensitivity is accounted for by its ability to prevent CORT from augmenting ER stress. Over the past several years, efforts by researchers have begun to uncover the interactions between GCs, the physiological stress response, and Se within the brain [34]. Studies on rodents exposed to various stress paradigms have shown that treatment with Se-containing organo-compounds can alleviate the associated neurological deficits, which include anxiety-like and depressive-like symptoms, as well as memory impairments [35,36]. Supplementation with sodium selenite was also found to reduce the oxidative damage caused to the rat brain by high-dose administration of the synthetic GC dexamethasone (DEX) [37]. Similarly, we have found that providing additional Se can counter the negative effects of GCs.

How might Se be working to counteract the effects of GCs on cellular physiology? One potential mechanism might be that Se is capable of altering the activity of the GCR. The function of the GCR can be affected by redox-dependent mechanisms, and one study found that selenite is capable of inhibiting the ligand binding activity of the GCR using in vitro preparations [38,39]. Additionally, experiments in isolated rat cardiomyocytes showed that an oxidative state increases the cellular response to GCs [40]. Conversely, several studies have demonstrated that reductive agents can promote GCR activity and an oxidative state can interfere with GCR activity in pituitary corticotroph cells [41]. Thus, the impact of redox state on the GCR may be cell type-specific. Additionally, the potential interactions between the redox-regulating properties of Se and GCR functionality remain under-investigated. Our current results suggest that, should such an interaction exist, Se, which promotes a reductive cellular environment, could potentially limit GCR activity.

Alternatively, our finding that Se supplementation can prevent CORT from potentiating ER stress in hypothalamic cells could indicate that CORT promotes ER stress through pro-oxidant means. In this case, Se would simply be limiting the down-stream oxidative impact of GC action rather than inhibiting GCR activity. There are, in fact, several previous reports of the ability of GCs to regulate selenoproteins, which may help with the interpretation of our results. One study by Wray et al., which focused on the obesogenic effects of GC exposure in mice, found that chronic CORT consumption alters the gene expression of selenoprotein P (*Selenop*) and iodothyronine deiodinase 2 (*Dio2*—also a selenoprotein) [4]. Work in human HEK-293 cells has shown that the GCR can prevent the transactivation of the *Selenop* gene [42]. A study using lung cancer cell lines found that GCs bind a pair of glucocorticoid response elements (GREs) located in the downstream regulatory region of the G*px3* gene, thereby increasing expression [43]. DEX was shown to induce adipogenesis in 3T3-L1 differentiated adipocyte-like human cells through an ER stress-dependent mechanism that involves the proteasomal degradation of SELENOS, an ER-resident selenoprotein [44]. Finally, a handful of studies have reported that GCs can decrease GPX1 protein and activity levels in hippocampal cells [7,45]. Thus, the protective effects we have observed in our experiments with Se supplementation could represent a prevention of GC-induced selenoprotein deficiency.

## 5. Conclusions

In summary, the data presented herein provides insight on the relationship between GCs and hypothalamic function in the context of energy homeostasis. Using an immortalized hypothalamic cell line, we have shown that repeated GC exposure can make cells more vulnerable to ER stress-induced disruptions in insulin signaling. Furthermore, increasing the availability of Se can alleviate the deficits in ER stress and insulin signaling caused by CORT. Our current data on Se and insulin signaling may, therefore, also provide a connection between chronic GC action and the associated neurological deficits. Future work will reveal the mechanistic intersections between GC and Se action and the underlying pathways through which Se can preserve insulin signaling in the brain.

## Data Availability

The data presented in this study are contained within the main text. Inquiries about data should be directed to the corresponding author.

## Abbreviations

Akt—Protein kinase B; CHOP—C/EBP homologous protein; CORT—Corticosterone; DEX—Dexamethasone; DIO2—Iodothyronine deiodinase 2; ER—Endoplasmic reticulum; GC—Glucocorticoid; GCR—Glucocorticoid receptor; HPA—Hypothalamic-pituitary-adrenal; ROS—Reactive oxygen species; Se—Selenium; Sec—Selenocysteine; SELENO-X—Selenoprotein-x; T2DM—Type II diabetes mellitus; VEH—Vehicle.
